# Self-Healing Asphalt: A Systematic Bibliometric Analysis for Identification of Hot Research Topics during the 2003–2018 Period

**DOI:** 10.3390/ma14030565

**Published:** 2021-01-25

**Authors:** Ricardo Abejón

**Affiliations:** Departamento de Ingeniería Química, Universidad de Santiago de Chile, Av. Libertador Bernardo O’Higgins 3363, Estación Central, Santiago 9170019, Chile; ricardo.abejon@usach.cl

**Keywords:** self-healing asphalt, heating, induction, rejuvenator, encapsulation, bibliometric analysis, research trends

## Abstract

The use of self-healing asphalt is a good option to extend the lifetime of roads and to improve the sustainability of pavement management systems. A bibliometric analysis based on the Scopus database was carried out to review the global research related to self-healing asphalt from 2003 to 2018 and to identify relevant quantitative characteristics from the research in this period. The results from this analysis revealed that the number of accumulated publications followed an exponential growth, which confirmed the relevance that this research topic has gained during the last years. The study revealed that China was the most productive country, followed by the Netherlands, where the most prolific institution is located: Delft University of Technology. Some important research features of the two main approaches most frequently used to develop asphalt mixtures with enhanced self-healing abilities (external heating and encapsulated rejuvenating agents) are compiled in this work.

## 1. Introduction

With the continuous increase in population and traffic demands, transport infrastructures have become essential for modern societies. These infrastructures allow the movement of goods, services, and people, which facilitate the connection of social and economic systems with the natural environment [1,2]. Therefore, transport infrastructures directly impact the three axes of sustainability, influencing the equilibria among economic, environmental, and social aspects, and consequently, the concern about sustainable management of transport systems has increased. Although transportation managers have traditionally focused only on the economic aspect of sustainability, an increasing number of transportation agencies have taken measures to embrace all principles of sustainability in pavement management practices [3,4]. Pavement management systems have been applied to roads since the 1970s to improve the maintenance of transport infrastructures, but a new impulse focused on sustainable pavement management has emerged, paying special attention to the trade-off between costs, environmental impacts, and social consequences of the investments in road networks [5,6,7]. In this new framework, infrastructure managers must consider a sustainable approach to the management of pavement maintenance to ensure technically appropriate solutions that are economically viable, environmentally sustainable, and socially acceptable, which requires complex solutions to encompass all these principles [8,9].

A sustainable approach in the planning and decision-making stages of a pavement management system must take into account that, as a consequence of the passing of time, roads age and deteriorate. Although roads are designed and built for use, it is necessary to take actions to increase their service cycle with minimal economic costs and environmental impacts [10]. During their service life, asphalt pavements are exposed to loads as a result of the combination of traffic and environmental actions (atmospheric conditions and precipitation) that imply deterioration of the pavements in diverse forms [11,12]. The main consequence is a progressive reduction in service level. These problems can be addressed by more frequent resurfacing and reconstruction of asphalt pavements but at significant costs to road agencies, additional related environmental impacts, and disturbances to users [13].

Cracks are one of the main manifestations of distress in asphalt pavements. The mechanisms involved in cracking of bituminous materials are complex and depend on a wide range of temperatures and loading conditions [14]. Asphalt mixtures are degraded due to oxidation caused by atmospheric conditions. This aging effect diminishes the material viscoelastic properties and makes asphalt binders stiffer and stiffer. Once high stiffness values are reached, the material becomes brittle and its capability to withstand repeated traffic loads is reduced and finally suffers cracking on microscopic and macroscopic scales [15,16,17]. In order to solve the irreversible damage caused by cracking failure of asphalt pavement, much research efforts have been conducted to promote crack closure at an early stage [18,19]. Although the intrinsic healing capacity of asphalt has been proven [20,21,22], its effect is clearly limited by field conditions and is not enough to compensate the degradation process. Therefore, the development of novel bituminous materials with improved self-healing has been investigated, focused on specific characteristics such as continuous damage sensing or autonomous repair [23]. Two main approaches must be highlighted among the ones studied to promote self-healing of cracks in bituminous materials: on the one hand, a reduction in the viscosity of bitumen by increasing its temperature through external heating and, on the other hand, the release of rejuvenators encapsulated in the asphalt mixture.

The amount of published scientific literature about self-healing asphalt available has increased rapidly during the last years. Bibliometric tools can be considered useful to manage all the information found from a bibliographic search. The term bibliometrics was first introduced by Pritchard, who explained that the term “deals with relationships among numbers of scientific papers, numbers of patents, amounts of exports and other quantities” [24]. In fact, bibliometrics refers to the research methodology employed in library and information sciences that applies quantitative analysis and statistics methods to describe the distribution patterns of publications according to some given categories. This methodological approach allows the organization and analysis of a high number of scientific documents and can be applied to the identification of important research trends, as demonstrated by several works in the engineering fields [25,26,27,28,29,30]. The aim of this work was the analysis from a bibliometric point of view of the scientific literature related to the research on self-healing asphalt published between 2003 and 2018 in the sources compiled in the Scopus database. The documents found were studied and evaluated according to several categories (annual outputs, leading countries, institutions, or main journals and languages) in order to determine the quantitative characteristics of the research on this topic worldwide and to identify the most relevant trends.

## 2. Data Sources and Methodology

Scopus was employed as the database for a bibliographic search of published scientific literature related to self-healing asphalt. This abstract and indexing database with links to full texts, which is managed by Elsevier, indexes content from 24,600 active titles and 5000 publishers, and is rigorously vetted and selected by an independent review board. Therefore, it is claimed to be the largest abstract and citation database of peer-reviewed literature, featuring smart tools to track, analyze, and visualize research literature [31]. It contains more than 70 million abstracts from all regions, including non-English titles when abstracts in English are provided. Consequently, more than 20% of titles on Scopus are published in languages other than English, including more than 40 local languages. Since more than 50% of Scopus content comes from outside North America (with relevant contributions from Europe, Latin America, and Asia), Scopus offers a broad worldwide coverage of peer-reviewed literature across the sciences, technology, engineering, and medicine (STEM) fields.

The online search within Scopus was completed in June 2019 after the selection of self-healing asphalt as keywords in the article title, abstract, and keyword fields of the search-engine. The keywords were introduced without quotations to find all papers that include all those words in any order. The search was limited to including 2018 as the last year considered in order to identify scientific documents related to the research on self-healing asphalt published until that date. The total number of documents recovered was 217.

The analysis of the scientific literature identified after a bibliographic search provides a valuable background for better understanding of the global research state in a subject like self-healing asphalt. In this context, the tasks included in this paper covered not only quantitative description of the publications (annual outputs, leading countries, institutions, and authors, or main journals and languages) but also a review of the most relevant research topics derived from study of the corresponding keywords, which can support the identification of current hot topics and the definition of future long-term research strategies.

## 3. Results and Discussion

### 3.1. Bibliometric Analysis of Research on Self-Healing Pavement

#### 3.1.1. Publication Year, Document Type, and Language of Documents

**Figure 1:**
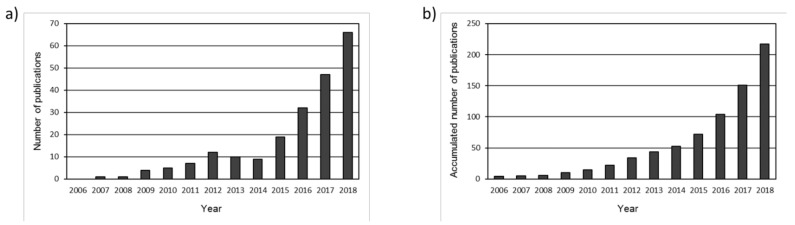
Annual (**a**) and accumulated (**b**) publication output.

The evolution of the annual distribution of publications and the total number of accumulated documents is shown in Figure 1. The earliest document found was published in 2003: a study that examined the self-heating properties of asphalt concrete when affected by various stresses inside the dam body [32]. In fact, until 2009, only 5 more documents were published regarding self-healing asphalt [33,34,35,36,37]. The data in Figure 1 give a clear idea about the regular increase in publication rates, especially since 2015. This great increase began a rampant evolution of the accumulated number of publications, with good fitting to an exponential model (R^2^ value equal to 0.995).

An analysis of the distribution of document types concluded that six different types were identified among the 271 publications. Articles (176) were the most frequent document type, comprising more than 81% of the publications, followed by conference paper, with 27 publications (12% of the total production). These figures imply that both types of documents summed up more than 93% of the found publications. The other less significant categories (joint contribution below 7%) included conference reviews (6 documents), reviews (6 documents), and a book and book chapter. These percentages were in agreement with the clear prevalence of articles over other types of documents in the scientific production by most engineering fields [38,39,40,41], although the data obtained by other bibliometric studies have pointed to the relevant contribution of conference papers in some civil engineering topics [42,43].

English was clearly the most frequent language in the documents found (85.7% of the publications were written in English). Only three other languages were found, with Chinese appearing as the second-most common language, with 29 documents (13.4%), and Spanish and Korean only represented by a unique publication each. Once again, as previously demonstrated by other bibliometric studies, English was the dominant language in the scientific literature regarding engineering topics, but in this case, the contribution was below 90%, a value registered by most bibliometric studies [44,45,46]. Therefore, the higher than usual contribution of documents written in Chinese pointed out the significant contribution of the research performed in China in the global scenario.

#### 3.1.2. Distribution of Output in Subject Categories and Journals

The distribution of research subjects is shown in Table 1, where the 8 most common categories are included, only the ones with at least 10 papers. Since subject categories are not exclusive, a document can be considered simultaneously in more than one research subject due to interdisciplinary research. Consequently, the sum of the number of documents in these subjects is higher than the total number of documents found in the bibliographic search and an equivalent result can be found when percentages are analyzed, with results above 100%. The ranking revealed that engineering was the dominant category, with a contribution percentage of 85.3% (185 papers). Moreover, materials science was an important category, since its contribution was 53.5% (116 papers). The collaboration between these two subjects covered a great number of the papers related to the research in topics related to self-healing asphalt, since the contribution of the third subject in the ranking (physics and astronomy) was less significant, with only 19 documents (below 9% contribution).

The distribution of these documents in sources is shown in Table 2. The corresponding values (year 2018) of Impact Factors (IF) from the Web of Science database and the SCImago Journal Rank (SJR) index from the Scopus database of the top 7 journals (only the ones with at least 5 publications) were also included. One journal clearly led the publication ranking with 53 documents (which represented 24.4% of the total number of papers): *Construction and Building Materials*. It is a peer-reviewed journal which covers the dissemination of innovative and original research and development in the field of construction and building materials and their application in new works and repair practice. Moreover, this journal shows the highest IF value (above 4), which demonstrates the high quality of the source. The second-most cited source is the Journal of Materials in Civil Engineering, which is focused on the development, processing, evaluation, applications, and performance of construction materials in civil engineering. However, this journal only contributed with 10 documents (4.6% contribution), far from the leading journal. The podium was completed by Jianzhu Cailiao Xuebao/Journal of Building Materials in the third position, a journal published by Tongji University in Chinese. The rest of the journals in Table 2 only contributed with 5 documents. Therefore, apart from the dominant leader of the most relevant journal in terms of high contribution and quality, the papers shared a high number of publications with relative low contribution values.

#### 3.1.3. Publication Distribution of Countries and Institutions

An analysis of author’s countries is compiled in Table 3, which shows the top 6 countries ranked by the number of total publications, only the ones with at least 10 publications. Once again, as in the case of subject categories, the country affiliation is a nonexclusive category, so a document can simultaneously be considered in more than one country as a result of international collaborations. The analysis revealed that China was clearly the most productive country, with 95 papers, which implies a contribution of 43.8%. This fact confirms the leadership of China in the research of self-healing asphalt, as previously pointed out by the relatively high number of documents written in Chinese. This leader country was followed by the Netherlands (43 papers and 19.8%, figures that implied that China doubled the production of the second-most productive country). Although the USA is usually found among the top contributors in most scientific and engineering fields [47,48], in this case, the American production (29 papers and 13.4% contribution) was ranked third, closely followed by the United Kingdom in fourth (24 papers and 11.1% contribution). Finally, the relevant production of a South American country like Chile must be highlighted, since it occupied the sixth position in the ranking, just after Spain.

When the top 8 institutions (only the ones that produced at least 10 documents) were identified, the important scientific production from these institutions located in the previously mentioned most relevant countries must be highlighted (Table 4). Unexpectedly, the ranking was not led by a Chinese institution, since Delft University of Technology was the most prolific institution, with 43 documents. This figure implies that this Dutch university monopolized all the scientific production from the Netherlands. Nevertheless, 5 Chinese affiliations appeared in Table 4 among the top 8 institutions. The ranking was completed with the University of Nottingham in fifth, with 22 papers, and the Universidad del Bio Bio in eighth, with 10 papers. Once again, as in the case of the Delft University of Technology, the contributions of the British and Chilean universities were critical, since they comprised 92 and 100% of the national productions, respectively. Surprisingly, American institutions did not appear among the most productive ones, a fact that is unusual in most research topics in engineering fields [49,50].

#### 3.1.4. Most Frequently Cited Papers

The 10 most cited documents identified after the bibliographic search are shown in Table 5. The number of citations augmented from 53 for the last document in the ranking to 134 for the leading article. Although further comments about the most important research trends will be included in the next section as a result of the analysis of the most frequently employed author keywords, the contents of the most cited documents can point to some relevant topics that gain attention from researchers investigating self-healing asphalt.

Three main topics dominate the ranking in Table 5. Firstly, investigations on the mechanisms that control self-healing and the development of models based on these mechanisms to represent the process were covered by 3 documents, including the most cited one [51,52,53]. On the one hand, systematic understanding of the healing mechanism is required to accurately model and predict the influence of self-healing on the performance of asphalt mixtures. On the other hand, assessment of the self-healing rates as a function of temperature is a valuable tool to determine the necessary heating times to obtain complete recovery of the original asphalt conditions. Secondly, induction heating of asphalt is a technique deeply investigated to promote the self-healing rates, and 5 articles covered this topic [54,55,56,57,58]. Induction heating is based on the addition of electrically conductive fibers or magnetically susceptible particles to the mixture of asphalt. A high-frequency alternating electromagnetic field can induce eddy currents in these added materials, which are heated, and the heat energy diffuses into the asphalt to enhance self-healing. The selection of the most adequate binders (fibers or particles), such as cast steel particles, steel wool, or graphite, and the optimal formulation of the asphalt mixture are key factors in the design of self-healing asphalts. Finally, the 2 articles that occupied the seventh and eighth ranks were focused on the use of microwave heating as an alternative to electromagnetic-induced heating to improve self-healing in asphalt [59,60].

#### 3.1.5. Analysis of Author Keywords

**Figure 2:**
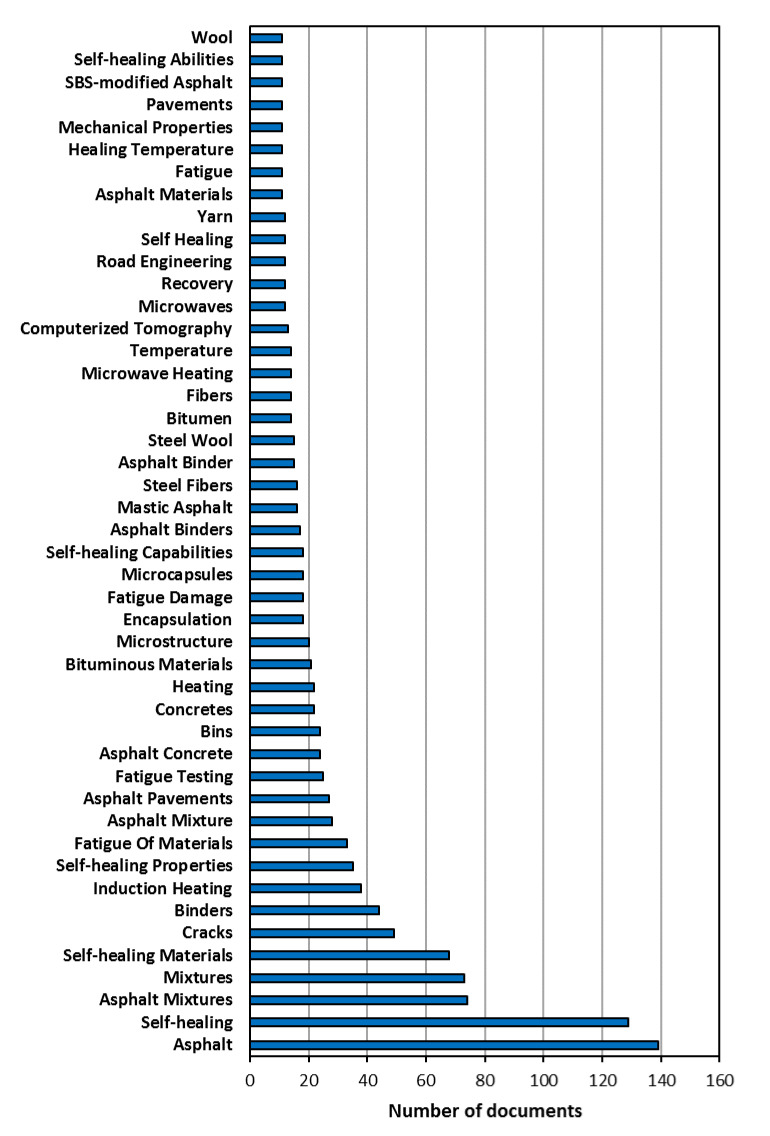
The top 46 most frequently used keywords.

The author keywords highlight the main focus of the research presented in a scientific document. An analysis of these keywords can be employed as a quantitative content breakdown to identify the most important topics and trends in different research fields [61,62]. Since an analysis of the most frequently used author keywords provides valuable information, this work applied this approach. The 46 most frequently used keywords, mentioned by at least more than 10 documents, are shown in Figure 2. Keywords that were directly related to the search terms were very frequent. For example, the first 5 keywords in the ranking (“asphalt”, “self-healing”, “asphalt mixtures”, “mixtures”, and “self-healing materials”) belonged to this category.

However, other keywords that were not directly related to the search terms were clearly more relevant in identifying information about the major research topics. The importance of the formulation of asphalt mixtures for enhancement of the self-healing properties must be highlighted, as demonstrated by the keyword “binders” (44 times), which occupied the sixth position in the ranking. As mentioned above during the analysis of the most cited papers, the selection of optimal fibers or particles as binders, as indicated by keywords like “steel fibers” (16 times), “steel wool” (15 times), or “fibers” (14 times), is a critical topic, specifically when the asphalt mixture is subjected to induction heating. In fact, “induction heating” was mentioned 38 times as a keyword and other examples of terms directly related to heat appeared in the ranking: “heating” (22 times), “microwave heating” (14 times), or “microwaves” (12 times). Apart from techniques based on induced healing by heating, another approach applied to improve the self-healing characteristics of asphalts mixtures is the addition of chemicals called rejuvenators. The mission of a rejuvenator is to diffuse into the aged binder and to restore its original molecular structure to extend the pavement service life [18]. The inclusion of a rejuvenator into the asphalt mixture via microcapsules offers the potential to achieve optimal performance. Therefore, the presence of keywords like “encapsulation” and “microcapsules”, appearing 18 times each, are clearly justified.

### 3.2. Review of Hot Research Topics in Self-Healing Asphalt

**Figure 3:**
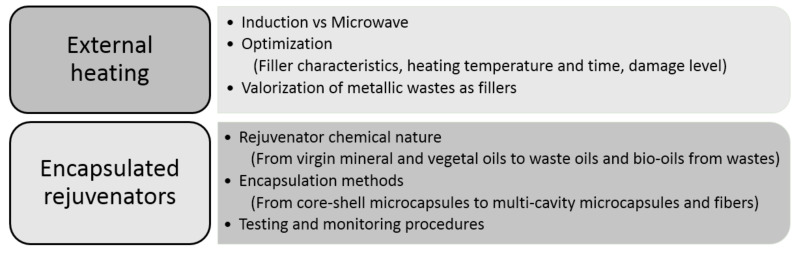
Main approaches and hot current research trends for self-healing asphalt.

Asphalt mixtures can repair minor damages caused by cracking at the microscale thanks to the development of self-healing bituminous materials, characterized by their ability to self-repair at least partially the damage suffered throughout their service life [63]. Two different approaches have been proven valid in promoting self-healing of cracks in bituminous materials: on the one hand, a direct reduction in the viscosity of bitumen by increasing its temperature through externally applied heating and, on the other hand, the use of encapsulated rejuvenating agents. Nevertheless, recent research has demonstrated the synergistic effects of rejuvenation and crack healing by external heating, which resulted in a longer life extension [64]. A schematic summary about the most important topics related to both approaches according to the found references is shown in Figure 3, and a more detailed analysis of each topic is carried out in the next subsections on the basis of the most significant documents.

#### 3.2.1. Induced Healing by Heating

Asphaltic materials have an intrinsic self-healing ability to repair their own damage during rest periods and to recover autonomously (at least partially) the strength lost. Temperature is a key factor affecting this self-healing ability: an increase in temperature not only increases the self-healing rate but also shortens the total time needed for full healing [18]. Unfortunately, the sun’s heating effect does not help enhance the self-healing properties of these materials. Tests performed with infrared lamps, which can be considered the best approach to simulated solar radiation [65], have demonstrated that there is an optimal infrared radiation energy for asphalt self-healing [66]. Once this threshold is overcome, further infrared radiation damages the materials. This fact explains the cracks that are not healed during warm seasons in roads exposed to very sunny environments. Furthermore, alternative heating mechanisms have been confirmed to be more energy efficient than infrared radiation. Induction heating is an illustrative example. By application of induction heating, the effect is concentrated only on the binder instead of heating the whole asphalt mixture like in the case of infrared heating. In fact, test samples exposed to induction heating healed in a few minutes, while equivalent samples exposed to infrared heating required several hours for healing [67]. In the case of infrared radiation, thermal energy is induced in the material through the upper face of the test samples and conducted downwards by the aggregates and bitumen. Therefore, the temperature of bitumen increases slower with infrared than with induction heating, since the critical energy required is higher because the aggregates must be also heated, and consequently, self-healing occurs at a slower rate.

The intrinsic self-healing properties of asphalt mixtures can be enhanced by means of induction heating. During induction heating, asphalt mixtures containing conductive particles are exposed to a high-frequency alternating electromagnetic field which induces eddy currents in materials that are electrically and magnetically susceptible. These metallic conductive particles are heated by the induced eddy currents and the heat energy diffuses into the whole mixture [18].

The healing process is more effective when these metallic fillers (steel wool is the most frequent one, but aluminum crumb has been also reported) are connected in closed-loop circuits [68,69]. Different filler contents with different lengths, quantities, and diameters of steel wool fibers have been evaluated, and as a recommendation, mixtures with around 5% content of short fibers with big diameters should be employed [56,70]. The influence of air void content has been investigated too. While dense mixtures obtained better healing with low energy, the maximum healing ratios were lower than those obtained by semi-dense and porous mixtures [67,71]. Although different types of bitumen have different adhesive and rheological properties and could affect the induction healing capacity of asphalt mixtures, the performed studies have concluded that the type of bitumen does not significantly affect the healing capacity of the asphaltic material [72,73]. Regarding induction heating speed and heating temperature, the optimal conditions are highly influenced by the type of asphalt mixture selected [58,74,75]. Simple models have been developed to represent the thermal heat flow equations for asphalt mixtures heated though induction energy, which have contributed to identifying and visualizing the main induction heating mechanisms [52]. The experimental tests have demonstrated that the fatigue life of induction-healing porous asphalt can be extended significantly by the application of induction heating, which could extend the service life of roads by at least 30% [55,73,76,77,78]. However, excessive heat application produces a detrimental effect on the healing rate, since the binder may completely melt and the properties of the material will be lost [57,66,79]. Even when this critical heat limit is not exceeded, the healing performance of asphalt mixture significantly decreases at every heating cycle [60,63].

Microwave radiation is a technique widely used as alternative heating, as it can heat rapidly throughout the material thickness, reducing processing times and saving energy. Metallic particles that may reflect microwave radiation and accelerate the increase in temperature can be added to asphalt mixtures. Thus, ferrous particles can be used to increase heating rates of asphalt mixtures because they can absorb and conduct more thermal energy than the other components [18]. Microwave heating of asphalt mixtures containing metal fibers (steel wool) is a promising technology for asphalt pavement rehabilitation by self-healing. In fact, the optimal steel wool content assessed is around ten times less than the one recommended for heating by electromagnetic induction, which in practice could mean an important reduction in costs [59]. Additionally, the amount of electricity used by microwave devices is much less than that required to produce a similar effect by electromagnetic induction, so microwave heating is more effective than induction heating in healing cracks in asphalt roads. The research in this field has determined that the penetration depth of microwaves can exceed 100 mm, which is useful for healing microcracks in the middle or bottom asphalt layers [80]. In order to evaluate the self-healing efficiency of asphalt mixtures exposed to microwave heating, the recovery rate of the stiffness modulus and the fatigue life extension ratio can be employed as healing indexes, and the influence of different factors, such as heating temperature, healing time, or damage level, has been investigated [81,82]. The heating time has been established as the most influential variable on the healing level reached by the asphalt mixtures exposed to microwave radiation. Less than a minute can be considered the optimal heating time to reach the highest healing levels with the lowest damage on the asphalt samples, since excessive microwave heating degrades bitumen and increases the porosity of asphalt mixture [60,83]. The self-healing process of asphalt mixtures by the induction or microwave heating methods showed poorer performances when the heat was employed in ice and snow melting processes. The moisture from melted snow and ice on crack surfaces prevents effective thermal healing [84].

Among the main disadvantages of asphalt induction or microwave heating, the increased economic costs and environmental impacts must be mentioned. At least, some recent proposals have identified a solution to the inconvenience caused by the need for metallic particles: the valorization of metallic wastes [85]. Several metallic wastes have been tested as fillers in asphalt mixtures, including steel slag, cutlery industry discards, metal fibers from old tires, and other steel shavings [86,87,88]. The research indicates that these metallic fillers are characterized by more irregular shapes than virgin steel wool fibers (mainly larger widths), but in fact, these wider elements can form electrically conductive channels, which improve the thermal conductivity and the specific heat capacity of asphalt mixtures [89,90]. However, these metallic wastes tend to form clusters during the mixing process, which limits the maximum volumetric content that can be added. The replacement of conventional coarse aggregates (such as granite or limestone) with steel slag is another promising option [91], since its presence not only can provide better healing results but also can improve the performance of the whole mixture [92]. The research tests have identified improved load–displacement relationships with higher ductile behavior, higher heating rates with enhanced energy conversion of microwave irradiation into more thermal energy, and other improvements [93,94,95]. However, examples demonstrating that the healing performance of these mixtures with metallic wastes was slightly lower have been published too [96]. Nevertheless, the metallic waste fillers appear to be a cheaper and more sustainable alternative since their specific use in base layers (the thickest ones) can contribute significantly to reducing the ecological impact of roads without increasing the costs and need for raw materials [85].

#### 3.2.2. Encapsulation of Rejuvenating Agents

The principle behind the use of encapsulated rejuvenators is that, when micro-cracks begin to form within the pavement system, they find a capsule in the propagation path. The fracture energy at the tip of the crack opens this capsule and releases the rejuvenating agent, which is mixed with the asphalt binder to seal the crack, thus preventing further propagation [97]. However, the main drawback of this approach is that it works only once, since once the rejuvenator is released from the capsule, it cannot be replenished. The effectiveness of an encapsulated rejuvenator depends on several factors, such as the rejuvenator chemical nature, the encapsulation technology, and the final interaction between the encapsulated rejuvenator and the aged bituminous material, which define their compatibility and the diffusion rate of the rejuvenator [98].

A wide variety of rejuvenators have been successfully employed in asphalt mixtures, including different types of vegetal oils, mineral oils, waste-derived oils, and more complex engineered products [99]. Rejuvenators must contain a high proportion of maltene constituents, which are required to keep asphaltenes dispersed [99]. Vegetal oils, such as soybean, sunflower, corn, cashew nut shell, or castor oils, have been reported to more effectively reduce the hardness of aged asphalt binders compared with rejuvenators based on mineral oils [100,101,102,103,104,105,106,107,108,109]. Moreover, the use of waste cooking oil is an eco-friendly solution due to its great performance as a rejuvenating agent [110,111,112,113,114,115]. Similarly, waste mineral oils can be reused as rejuvenators without further refinement [115], although vegetal oil-based rejuvenators usually require lower doses than the ones based on petroleum to achieve similar performance [116,117,118,119,120,121,122]. Another green option to produce rejuvenators is the use of organic waste to obtain bio-oil [123,124,125,126,127,128]. Although the road performance of bio-oil-rejuvenated asphalt mixtures varies greatly (it depends on the raw biomass treated, the bio-oil production process, etc.), this alternative has better environmental benefits when compared to the use of virgin mineral oils [129,130,131,132,133,134,135,136]. The rejuvenators can be used in combination with additives like polymers or other modifiers to improve the overall performance and the manageability during the encapsulation process [137,138].

The encapsulation technologies effectively applied to many industrial sector (pharmaceuticals, food, etc.) can be used to produce microcapsules for rejuvenators in asphalt self-healing applications [23]. The chemical encapsulation methods based on polymerization are the most frequently selected technology [139,140,141], but alternatives based on gelation or coacervation have been successfully implemented [142,143,144]. The optimal size of the microcapsules is critical, and it must be controlled during encapsulation. On the one hand, the size of the microcapsule needs to be less than 50 µm to avoid being squeezed or pulverized during asphalt pavement mixing and compaction processes [97]. On the other hand, microcapsules below 10 µm are not suitable for self-healing since they do not contain sufficient rejuvenators [145]. Regarding the morphology of the capsules, three main groups can be mentioned. Firstly, core-shell microcapsules consist of a liquid core (the rejuvenating agent) surrounded by a double shell that encapsulates the rejuvenator. The most common material used as shell material is a commercial prepolymer (melamine-formaldehyde) modified by methanol [144,145,146]. Secondly, beads based on calcium alginate are the most representative polynuclear capsules. These alginate beads are formed by letting an emulsion of sodium alginate and rejuvenator drop into a hardening solution of calcium chloride [140,142,147,148]. There are two main advantages of these multi-cavity elements. They present a better structural reinforcement, which allows for their integrity during asphalt manufacturing processes, and the beads do not release all of the rejuvenator when the first crack reaches due to their compartmented structure, so they provide multiple crack-healing and long-term healing [23,149]. Finally, recent research efforts have paid special attention to the development of encapsulated fibers [129,150,151,152,153]. These systems can better ensure the release of rejuvenator into cracks, since the crack has a higher probability to pass through the fiber networks due to their dimensions. In addition, they can supply larger volumes of the rejuvenator.

Different standard and innovative procedures have been adapted and developed to quantify the self-healing capability in bituminous materials by the action of encapsulated rejuvenators, and a full review of this topic can be consulted in [23]. The self-healing capability is commonly measured through a healing level index assessed by testing a sample before and after the healing process. Both static and dynamic mechanical tests are employed to characterize the most relevant performance-related properties (stiffness, fatigue, rutting, etc.) of bituminous materials [154]. An alternative physicochemical approach is based on characterization through the analysis of physical properties, such as penetration, softening point, or viscosity, of the bituminous material in different states (virgin, aged, and rejuvenated). Another group of tests consists of direct observation by microscopy techniques to evaluate the self-healing process. Observation of the rejuvenator movement (diffusion and capillarity phenomena) and monitoring of the fracture morphology of the material before, during, and after the micro self-healing process provide valuable information to better understand the self-healing mechanisms and the influence of different factors [155]. Basically, the rejuvenator diffusion process can be summarized in three steps: 1) leaking of the rejuvenator from the broken microcapsules, 2) rejuvenator flowing by capillary forces, and 3) rejuvenator diffusion due to a concentration gradient [156].

## 4. Conclusions

An overview of the research related to self-healing asphalt was presented with information related to annual publications, document types, languages, countries, institutions, categories, journals, and research emphases, and tendencies. The earliest document found was published in 2003, and until 2009, production was very limited (only 5 additional documents). After this year, the production rate experienced a great increase, and the evolution of the accumulated number of papers followed an exponential trend. Articles were the most frequent document type, comprising more than 81% of the publications (176 papers), followed by conference papers, with 27 publications (12% of the total production). The study revealed that China was the most productive country, with 95 documents (43.8% contribution), followed by the Netherlands (43 papers and 19.8%). The relevant production of a South American country like Chile must also be highlighted, since it occupied the sixth position. The leading organization was a Dutch institution, Delft University of Technology (43 documents and 15.9% contribution), followed by three Chinese institutions.

The research related to asphalt self-healing induced by an external source of heat has been comparing the performance of electromagnetic induction and microwave heating in order to identify the most effective and efficient process. Although the use of microwave heating seems more effective in healing cracks in asphalt, further work is still required to define the optimal conditions, such as the filler characteristics and content, the heating temperature and time, or the most adequate damage level to apply the treatment. In addition, the valorization of metallic wastes to be used as fillers in asphalt mixtures can significantly reduce the environmental and economic impacts of this technical solution.

Regarding the use of an encapsulated rejuvenator for self-healing asphalt, research efforts have paid great attention to the chemical nature of the rejuvenator. The replacement of virgin vegetal and mineral oils by waste oils and bio-oils derived from organic wastes implies a significant improvement in environmental and economic terms without technical drawbacks. The multi-cavity microcapsules produced by new encapsulation techniques present advantages when compared to traditional core-shell microcapsules (better integrity during the asphalt manufacturing processes and long-term healing), while encapsulated fibers ensure better release of larger volumes of the rejuvenator into the cracks. Different procedures have been proposed to quantify the self-healing capability by encapsulated rejuvenators and to obtain better understanding of the self-healing mechanisms.

This identification of the hot topics on self-healing asphalt research should be useful in providing information to research organizations to evaluate the past research focuses, the projects they were supporting, and the issues that are still pending to be solved.

## Figures and Tables

**Table 1:** The top 8 most popular subject categories.

Ranking	Subject Categories	Documents	Percentage (%)
1	Engineering	185	85.3
2	Materials Science	116	53.5
3	Physics and Astronomy	19	8.8
4	Chemistry	18	8.3
5	Chemical Engineering	17	7.8
6	Energy	16	7.4
7	Social Sciences	11	5.1
8	Environmental Science	10	4.6

**Table 2:** The top 7 most productive journals.

Ranking	Journal	IF 2018 (WoS)	SJR 2018 (Scopus)	Documents	Percentage (%)
1	Construction and Building Materials	4.046	1.522	53	24.4
2	Journal of Materials in Civil Engineering	1.984	1.055	10	4.6
3	Jianzhu Cailiao Xuebao/Journal of Building Materials	-	0.232	9	4.1
4	Materials and Structures/Materiaux et Constructions	2.548	1.525	5	2.3
5	Rilem Bookseries	-	0.249	5	2.3
6	Road Materials and Pavement Design	1.980	0.963	5	2.3
7	Transportation Research Record	0.748	0.537	5	2.3

**Table 3:** The top 6 most productive countries.

Ranking	Country	Documents	Percentage (%)
1	China	95	43.8
2	The Netherlands	43	19.8
3	The United States	29	13.4
4	The United Kingdom	24	11.1
5	Spain	14	6.5
6	Chile	10	4.6

**Table 4:** The top 8 most productive institutions.

Ranking	Institutions	Country	Documents	Percentage (%)
1	Delft University of Technology	The Netherlands	43	15.9
2	Wuhan University of Technology	China	31	11.4
3	Ministry of Education China	China	27	10.0
4	Tongji University	China	25	9.2
5	University of Nottingham	The United Kingdom	22	8.1
6	Chang’an University	China	13	4.8
7	Chongqing Jiaotong University	China	11	4.1
8	Universidad del Bio Bio	Chile	10	3.7

**Table 5:** The top 10 most cited papers.

Ranking	Articles	Times Cited
1	Title: “Self-healing of open cracks in asphalt mastic” Authors: García, A. Source: Fuel Published: 2012	134
2	Title: “Electrical conductivity of asphalt mortar containing conductive fibers and fillers” Authors: García, A., Schlangen, E., Van de Ven, M., Liu, Q. Source: Construction and Building Materials Published: 2009	111
3	Title: “Induction healing of asphalt mastic and porous asphalt concrete” Authors: Liu, Q., García, A., Schlangen, E., Van den Ven, M. Source: Construction and Building Materials Published: 2011	81
4	Title: “Use of molecular dynamics to investigate self-healing mechanisms in asphalt binders” Authors: Bhasin, A., Bommavaram, R., Greenfield, M.L., Little, D.N. Source: Journal of Materials in Civil Engineering Published: 2011	76
5	Title: “Induction healing of dense asphalt concrete” Authors: García, A., Bueno, M., Norambuena-Contreras, J., Partl, M.N. Source: Construction and Building Materials Published: 2013	69
6	Title: “Evaluation of the induction healing effect of porous asphalt concrete through four-point bending fatigue test” Authors: Liu, Q., Schlangen, E., Van De Ven, M., Van Bochove, G., Van Montfort, J. Source: Construction and Building Materials Published: 2012	62
7	Title: “Heating asphalt mixtures with microwaves to promote self-healing” Authors: Gallego, J., Del Val, M.A., Contreras, V., Páez, A. Source: Construction and Building Materials Published: 2013	59
8	Title: “Self-healing of asphalt mixture by microwave and induction heating” Authors: Norambuena-Contreras, J., García, A. Source: Materials and Design Published: 2016	55
9	Title: “Induction healing of fatigue damage in asphalt test samples” Authors: Menozzi, A., Garcia, A., Partl, M.N., Tebaldi, G., Schuetz, P. Source: Construction and Building Materials Published: 2015	54
10	Title: “A simple model to define induction heating in asphalt mastic” Authors: García, A., Schlangen, E., Van de Ven, M., Liu, Q. Source: Construction and Building Materials Published: 2012	53

## Data Availability

Data sharing is not applicable to this article.
